# Small GTPases of the Rab and Arf Families: Key Regulators of Intracellular Trafficking in Neurodegeneration

**DOI:** 10.3390/ijms22094425

**Published:** 2021-04-23

**Authors:** Alazne Arrazola Sastre, Miriam Luque Montoro, Hadriano M. Lacerda, Francisco Llavero, José L. Zugaza

**Affiliations:** 1 Achucarro Basque Center for Neuroscience, Science Park of the UPV/EHU, 48940 Leioa, Spain; alazne.arrazola@ehu.eus (A.A.S.); miriamluquem@gmail.com (M.L.M.); 2 Department of Genetics, Physical Anthropology and Animal Physiology, University of Basque Country UPV/EHU, 48940 Leioa, Spain; 3 Three R Labs, Science Park of the UPV/EHU, 48940 Leioa, Spain; hadrilac@gmail.com; 4 Hospital 12 de Octubre Research Institute (i+12), 28041 Madrid, Spain; 5 IKERBASQUE, Basque Foundation for Science, 48013 Bilbao, Spain

**Keywords:** Rab GTPase, Arf GTPase, small GTPase, Alzheimer, Parkinson, neurodegeneration, membrane trafficking, vesicle, transport

## Abstract

Small guanosine triphosphatases (GTPases) of the Rab and Arf families are key regulators of vesicle formation and membrane trafficking. Membrane transport plays an important role in the central nervous system. In this regard, neurons require a constant flow of membranes for the correct distribution of receptors, for the precise composition of proteins and organelles in dendrites and axons, for the continuous exocytosis/endocytosis of synaptic vesicles and for the elimination of dysfunctional proteins. Thus, it is not surprising that Rab and Arf GTPases have been associated with neurodegenerative diseases such as Alzheimer’s and Parkinson’s. Both pathologies share characteristics such as the presence of protein aggregates and/or the fragmentation of the Golgi apparatus, hallmarks that have been related to both Rab and Arf GTPases functions. Despite their relationship with neurodegenerative disorders, very few studies have focused on the role of these GTPases in the pathogenesis of neurodegeneration. In this review, we summarize their importance in the onset and progression of Alzheimer’s and Parkinson’s diseases, as well as their emergence as potential therapeutical targets for neurodegeneration.

## 1. Introduction

Eukaryotic cells constantly receive information from the extracellular medium by the binding of growth factors, hormones, peptides, and ions to specific receptors. This binding triggers the transmission of a message through signaling cascades in the cytoplasm to induce a precise biological response [1]. One of the central elements responsible for the diffusion of this message are the small guanosine triphosphatases (GTPases) of the Ras superfamily. These small GTPases participate in signaling cascades that control a wide range of cell responses, such as proliferation, differentiation and apoptosis [2,3].

The small GTPases are molecular switches that can be found in two states: an inactive state in which the small GTPase is bound to GDP, and an active state in which it is bound to GTP. The process by which the GTPase changes from the inactive to the active state is known as the GTPase activation cycle. Three main molecules control the activation/deactivation cycle. The guanine exchange factors (GEFs) are in charge of activating the GTPase by favoring the release of GDP and the binding of GTP. The GTPase activating proteins (GAPs), on the contrary, are responsible for the inactivation of the GTPase by inducing the intrinsic GTPase activity that results in the hydrolysis of the GTP. Finally, guanine nucleotide dissociation inhibitors (GDIs) prevent the dissociation of the GDP from the GTPase, therefore maintaining the GTPase in an inactive state [4,5]. Moreover, small GTPases can also be regulated by post-translational modifications that permit their binding to either specific proteins or membranes. Thus, they can be farnesylated, geranylgeranylated or palmitoylated in their C-terminal region and myristoylated in their N-terminal region [5,6]. 

The Ras superfamily of small GTPases is divided into five families: Ras, Rho, Rab, Arf, and Ran [2,3]. The Ras family is specialized in the control of cell growth and metabolism. Additionally, Ras family GTPases cooperate with the Rho family to regulate the cell cycle, gene expression and cell transformation. Apart from those functions, the Rho family of GTPases are responsible for the actin cytoskeleton organization, whereas the Rab and the Arf families control the intracellular traffic of vesicles and membranes and the formation and intracellular transport of vesicles, respectively. Last, the GTPases of the Ran family are in charge of the nucleocytoplasmic transport [2,3,5,7]. 

**Figure 1:**
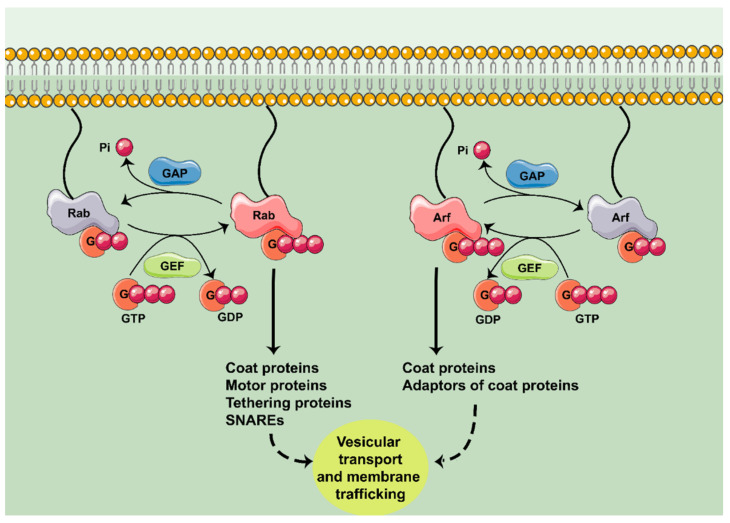
Rab and Arf GTPases and their role in membrane trafficking. Rab and Arf GTPases are activated by guanine exchange factors (GEF). Once activated, they interact with their effector molecules. Rab GTPases can interact with coat proteins, motor proteins, tethering proteins and SNAREs; Arf GTPases interact with coat proteins and adaptors. This results in the control of vesicle transport and membrane trafficking. The cycle culminates by the inactivation of the GTPase by GTPase activating proteins (GAP). G: guanosine.

Most of the intracellular compartments, such as the nucleus, mitochondria or the Golgi apparatus (GA), are separated by membranes. Thus, eukaryotic cells require specific mechanisms for the traffic between these organelles. Furthermore, coordinated membrane trafficking between different cell types is needed in multicellular organisms [8]. The Rab GTPases, the largest family of the Ras superfamily, are key regulators of vesicle sorting and membrane trafficking. They can control this traffic by interacting with effector molecules such as the coat proteins (COPI, COPII, and clathrin), motor proteins (kinesins and dyneins), tethering complexes (early endosome antigen 1 (EEA1), Golgins, exocyst and the homotypic fusion and protein sorting (HOPS) complex), and SNAREs [8]. Conversely, Arf GTPases participate in vesicle formation, especially in the GA [9], but they are also present in the plasma membrane, endosomes and lipid droplets [9]. To regulate vesicle formation, like Rab, the Arf GTPases interact with effector molecules such as the coat proteins and their adaptors (COPI, Golgi-localized γ-ear containing Arf-binding proteins (GGA), and Munc18-interacting proteins (MINT)). Therefore, the Rab and Arf families of GTPases regulate the endomembranes system (Figure 1). 

Membrane trafficking plays an important role in neurons. Neurons have a specific morphology that requires constant membrane trafficking between axons and dendrites to maintain synaptic function [8]. This enables synaptic transmission, the correct distribution of membrane receptors and precise organelle and protein composition in dendrites and axons [8]. Synaptic function demands a continuous flux of membranes, as synaptic vesicles are constantly subjected to exocytosis and endocytosis. Additionally, proteins must be transported between the axon, dendrites and cell body to transmit the signaling message or to be degraded. Besides, the retrograde transport of late endosomes and autophagosomes allows the removal of dysfunctional proteins, which is important for the correct neuronal function and survival. Hence, membrane trafficking is involved in all of the aspects of neuronal function, and its dysfunction has been linked to neurodegeneration [8].

Neurodegeneration consists the progressive loss of specific subsets of neurons [10]. The main neurodegenerative diseases are Alzheimer’s disease (AD) and Parkinson’s disease (PD). AD is the most common form of dementia [11]. It is characterized by the progressive loss of neurons that results in the loss of memory and cognitive functions. The principal hallmarks of the disease are the extracellular amyloid-β (Aβ) plaques and the intracellular accumulation of neurofibrillary tangles (NFTs), formed by pTau aggregation. Despite being those the classical features, the molecular pathology of AD is not completely understood. On the one hand, the amyloidogenic processing of the amyloid precursor protein (APP) that leads to the generation of Aβ peptides occurs in the intracellular compartments that require endocytic trafficking. Under physiological conditions, the APP is processed by the β-secretase (BACE1) in the Rab5-positive early endosomes, giving rise to β-cleavage C-terminal fragments (β-CTFs). Such fragments are then processed in late endosomes or the trans-Golgi network (TGN) to produce Aβ peptides [12]. This highlights the importance of these GTPases and membrane trafficking in AD pathology. Besides, various genes related to endocytic trafficking have been associated with the risk of developing AD [12]. For instance, a low expression of phosphatidylinositol binding clathrin assembly protein (PICALM) has been described in AD, which plays an important role in the internalization, trafficking and clearance of Aβ peptides [12,13].

Regarding PD, it is the second most common neurodegenerative disease. It is characterized by the accumulation of Lewy bodies, formed by α-synuclein (α-syn) aggregation, and by the selective degeneration of dopaminergic neurons of the substantia nigra pars compacta [14]. This results in disabilities in movements, including resting tremor and muscular rigidity. Mutations in α-syn, in PTEN-induced putative kinase 1 (PINK1) and in leucine-rich repeat kinase 2 (LRRK2) have been associated with the risk of developing PD [14]. Apart from these mutations, mutations in the Rab39B GTPase have been related to the development of this disease [15]. Rab39B controls the trafficking of the GluA2 subunit of the AMPA receptor and it is exclusively expressed in neurons [15]. Furthermore, various GTPases have been associated with the defects in membrane trafficking that appear owing to α-syn accumulations [15]. Thus, in the same way as in AD, these GTPases and membrane trafficking are related to PD pathology.

In summary, small GTPase-dependent membrane trafficking plays an important role in the nervous system, and dysregulations of such processes have been correlated with neurodegenerative diseases such as AD and PD (Table 1). As a result, in a similar fashion to the Ras and Rho families [5], the Rab and Arf family of GTPases have emerged as therapeutical targets for these pathologies.

## 2. Rab GTPases in Neurodegeneration

Small GTPases of the Rab family are responsible for controlling vesicular transport and membrane trafficking. They regulate all the steps of this transport; the biogenesis of carriers, their movement across the cytoskeleton, and their tethering in the target membranes [38,39].

As the rest of the members of the Ras superfamily, the activity of Rab GTPases is regulated by GEFs, GAPs, and GDIs. Two main families of RabGEFs have been described. The first is the DENN domain-containing family of GEFs, which can activate different Rab GTPases [40]. DENN is the catalytic domain that interacts directly with Rab GTPases [40]. The second is the Vps9 domain-containing family of GEFs, which are specific for Rab5 GTPases [41]. Apart from these two families, other proteins have been shown to act as GEFs for Rab GTPases, such as the TRAPP I and Mon1/Ccz1 complexes, which are GEFs for Rab1 and Rab7, respectively [41].

On the other hand, whereas GEFs share low sequence homology amongst them, Rab GAPs are classified into a unique family, the Tre-2/Bub2/Cdc16 (TBC)-domain GAPs. In humans, there is a single GAP that does not contain this TBC domain, the Rab3GAP complex [41]. Unfortunately, GEFs and GAPs for several of the Rab GTPases have not been described yet [41,42].

Apart from being regulated by their activation state (GDP-bound/GTP-bound), Rab GTPases can be found both in their active and inactive state in the cytosol or membranes. This localization is controlled by prenylation of the C-terminal cysteine residues. Once the vesicular transport is complete, Rab GTPases must be recycled and transported from membranes back to the cytosol. GDIs bind to prenylated and inactive (GDP-bound) Rab GTPases and then, the GTPases are removed from the membrane. Thus, the recycling of Rab GTPases is only accomplished once the vesicular transport is complete and the GTPase is inactivated by a GAP [41]. Nevertheless, prenylation is not the unique post-translational modification that regulates Rab GTPases. Some Rabs can be phosphorylated by kinases such as p34cdc2 or the PD-related kinase LRRK2 [41,43]. The pathogenic variants of LRRK2 associated with PD result in an increase in such phosphorylation. This post-translational modification occurs in the switch II domain, which is crucial for the GTPase interaction with its regulators. Specifically, phosphorylation reduces the interaction of the GTPase with its regulators [43,44].

As previously mentioned, Rab GTPases control all of the key steps of vesicular transport and membrane trafficking, due to their ability to interact with different effector molecules [45]. For the cargo selection, budding, and coat formation, Rab GTPases interact with proteins such as TIP47 or retromer. For instance, Rab9-GTP interacts with TIP47 in late endosomes, increasing TIP47 affinity towards the cargo that must be transported [46]. TIP47 recognizes the cytoplasmic domains of mannose 6-phosphate receptors (MPR), activating the transport from endosomes to the Golgi complex [46]. Another example is the interaction of Rab7 with the retromer complex to mediate the endosome-to-Golgi complex transport [47].

Regarding the regulation of vesicular transport, Rab GTPases interact with motor proteins such as kinesins and dyneins. Kinesins and dyneins are ATPases that use ATP hydrolysis to induce conformational changes that generate the motile force to move the cargo towards the plus-end and the minus-end of microtubules, respectively [48]. The Rab GTPases such as Rab3A, 6, 8A, 10, 11A, 14, 27A, and 39B interact with myosin type V to transport organelles and vesicles through actin filaments [49]. For instance, Rab27A interacts with myosin type V and melanophilin, forming a ternary complex to transport melanosomes towards actin filaments [50]. For the control of the uncoating and tethering of vesicles, Rab GTPases associate with proteins such as TRAPP, Exocyst, or p115/Golgins. One example is the interaction of Rab1 with p115, which is a tethering protein that induces the formation of the SNARE complex and stimulates the docking of COP I-coated vesicles in Golgi membranes [51]. Moreover, Rab1 also interacts with other tethering factors such as GM130 and GRASP65 to facilitate the fusion of the Golgi membranes vesicles [52]. GM130 is then responsible for the maintenance of the Golgi structure [52]. It is known that Rab GTPases interact with proteins such as Sro7 and Rabenosyn-5 [45]. For example, Rab8 interacts with Sro7, regulating SNARE proteins functions in the fusion of vesicles to the cell membranes while Rabenosyn-5 serves as a nexus between Rab and hVPS45 [53,54] by bringing together Rab4 and/or Rab5 and hVPS45-associated Rabenosyn-5, which then binds SNAREs.

In conclusion, Rab GTPases are the master regulators of cargo selection, formation, transport, docking and the fusion of vesicles with target membranes. Taking into account the importance of membrane trafficking in the nervous system, neurons have developed specific mechanisms to control the transport of proteins, organelles and receptors through long distances in axons and dendrites. Rab GTPases regulate the recycling, exocytosis and endocytosis of synaptic vesicles; the liberation of neurotransmitters; the traffic of receptors; and the anterograde and retrograde axonal transports [15]. What is more, they are also involved in the branching and morphogenesis of dendrites, neurite growth and neuronal migration during development. Considering the importance of such processes, the dysregulation of Rab GTPases has been related to various neurodegenerative diseases such as AD, PD, amyotrophic lateral sclerosis (ALS), and Charcot–Marie–Tooth (CMT) [8,15].

**Figure 2:**
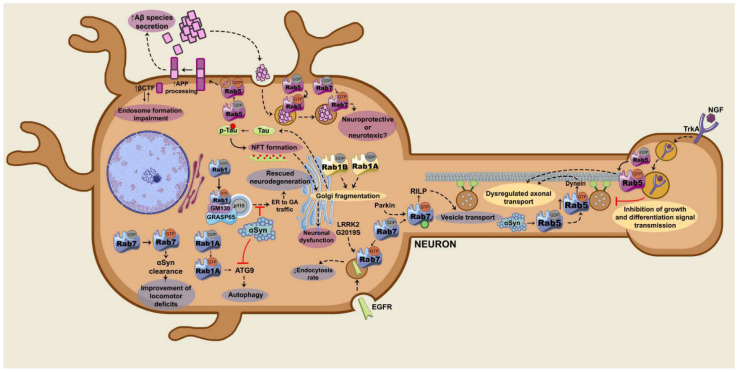
Scheme of the signalling pathways controlled by the small guanosine triphosphatases (GTPases) of the Rab family that are dysregulated in Alzheimer’s disease (AD) (purple), in Parkinson’s disease (PD) (blue) or in both (yellow). Silencing Rab1 induces Golgi fragmentation, while Rab1 overexpression can rescue it in both diseases. Rab1-regulated ER to GA traffic is inhibited by α-syn in PD. α-syn also alters Rab1A/Atg9 axis and consequently reduces the autophagosome formation. Regarding Rab5, its overactivation could alter the axonal transport of growth signals in AD. In PD, α-synuclein (α-syn) overexpression results in an activation of Rab5, which in turn interacts with dynein and dysregulates the axonal transport. Rab5 also participates in amyloid precursor protein (APP) processing, as well as in amyloid-β (Aβ) clearance in coordination with Rab7. This Aβ internalization by Rab5/Rab7 is considered as neuroprotective according to some studies, whereas it is considered as neurotoxic according to others. Rab7 favors the clearance of α-syn aggregates. LRRK2G2019S alters the endocytosis rate, which can be reverted by constitutively active Rab7 overexpression. Parkin ubiquitinates Rab7 and regulates vesicle transport via the Rab7/Rab-interacting lysosomal protein (RILP) axis. βCTF: β-cleavage C-terminal fragments; ER: endoplasmic reticulum; GA: Golgi apparatus; NFT: neurofibrillary tangle; NGF: nerve growth factor.

In AD, various Rab GTPases are involved in the transport of proteins related to the pathology, such as Tau, APP, BACE1, α-secretase, γ-secretase, and Aβ peptides. Furthermore, the expression of these GTPases is altered in the post mortem AD brain [55]. Regarding PD, these GTPases control the transport of α-syn [56]. Additionally, Rab GTPases could be mediating the toxicity caused by the LRRK2 kinase in PD [57]. As mentioned above, some Rab GTPases are substrates of LRRK2 and the dysregulation in this phosphorylation has been described to induce neurotoxicity and the degeneration of dopaminergic neurons in vivo [57,58]. Hereunder, we describe the specific roles of the main Rab GTPases in the onset and progression of AD and PD (Figure 2).

### 2.1. Rab1

Rab1 GTPases control the bidirectional transport between the endoplasmic reticulum (ER) and the GA, as well as the formation, integrity and recycling of Golgi membranes [38,59]. The Rab1 family is composed of two isoforms: Rab1A and Rab1B. The GEF for both isoforms is TRAPP I. TRAPP I is a complex of proteins that activates Rab1 and is involved in the ER–Golgi transport [41,60]. On the other hand, the molecule responsible for the inactivation of Rab1 is TBC1D20 GAP [41,61].

Many studies highlight the importance of Rab1, as well as its regulators, in the maintenance of the integrity of Golgi membranes. The overexpression of dominant-negative forms of Rab1A and Rab1B, the depletion of both GTPases, and the overexpression of TBC1D20 GAP induce the fragmentation of the GA [38].

#### 2.1.1. Rab1 and the ER–Golgi Traffic

Rab1 controls the transport between the ER and the GA, as it can interact with p115 and GM130-GRASP65 to favor the fusion of ER-vesicles in the GA [62,63,64]. Through its interaction with these effector molecules, Rab1 governs the formation, integrity and recycling of GA membranes. On the one hand, Rab1 interacts with p115 protein, which is a vesicle tethering factor, to control this ER–GA traffic [65]. On the other hand, when Rab1 associates with the GM130-GRASP65 complex in the GA, it regulates the stacking of the GA and vesicle binding [66,67]. GM130 is responsible for the integrity of Golgi membranes [52]. Moreover, it is believed that p115 can interact with GM130-GRASP65 for ER-vesicle fusion in the GA [62,64].

Furthermore, Rab1 also controls the retrograde transport between GA and the ER. To do so, the GTPase interacts with GBF1, a GEF for the Arf1 GTPase that is involved in the biogenesis of COP I vesicles [68,69].

Although the role of Rab1 in the ER–GA traffic in AD pathogenesis is not yet clear, it has been described that this GTPase could prevent the loss of dopaminergic neurons in PD [19]. In PD, one of the possible mechanisms by which α-syn could be inducing neurodegeneration is by inhibiting the ER–GA traffic [19]. It has been described that wild type (WT) α-syn, as well as the mutant α-syn^A53T^ that causes early-onset PD, block the ER–GA traffic, although α-syn^A53T^ initiates this blockage more rapidly than the WT. Cooper and collaborators have demonstrated that this α-syn-induced toxicity is prevented in the presence of Rab1 [19]. In fact, in *Drosophila melanogaster* (*D. melanogaster*), *Caenorhabditis elegans* (*C. elegans*) and primary cultures of rat neurons that express WT α-syn or α-syn^A53T^, the expression of Rab1 rescued the loss of dopaminergic neurons [19]. These data suggest that Rab1 could play a protective role in the control of ER–GA traffic and, therefore, could prevent neurodegeneration in PD.

Rab1 and its function in the control of ER–GA traffic are also related to ALS. The mutations in SOD1, TDP-43 or FUS proteins that cause this neurodegenerative disease result in a mislocalization of Rab1, as well as in an impaired ER–GA transport and increased ER stress [8]. Rab1 overexpression, on the contrary, exerts a protective role against this stress [8,21].

#### 2.1.2. Rab1 and the Integrity of the GA

Apart from the classical hallmarks of AD and PD pathologies, it has been described that neurons present a fragmented GA in both cases [70]. This fragmentation has been attributed to various causes, such as the presence of protein aggregates in the cytoplasm, alterations in the cytoskeleton or the malfunction of intracellular trafficking. In this regard, Martínez-Menárguez et al. state that the main reason for GA fragmentation in neurodegenerative diseases are the alterations in the intracellular transport [70].

Several studies have demonstrated that in neurodegenerative pathologies, Rab1-mediated traffic dysregulation induces GA fragmentation [16,17,70]. In the case of AD, those GA alterations have been ligated to pTau levels [71,72]. In 2014, the study of Jiang and collaborators revealed that GA fragmentation preceded Tau hyperphosphorylation [71]. According to them, GA fragmentation promotes Tau phosphorylation through the activation of cyclin-dependent kinase-5 (cdk5) and ERK.

Moreover, in AD patients, neurons exposed to NFTs present bigger defects at the Golgi in comparison to neurons without NFT [72]. Neurons that accumulated intermediate levels of pTau before NFT formation showed intermediate defects in the GA [72]. This supports that the progressive accumulation of pTau is associated with structural alterations in the GA. According to Antón-Fernández and collaborators, these alterations could affect the processing and trafficking of proteins, and therefore, they could contribute to neuronal dysfunction in AD [72].

Furthermore, the overexpression of Rab1A in HeLa cells expressing human Tau and primary neurons of rat cortex prevented GA fragmentation, whereas the silencing of the GTPase by siRNA induced its fragmentation [16,17]. They observed that Rab1A was co-localizing with GM130 in primary cultures of neurons from the rat cortex [16]. Another effect of Rab1A silencing was the up-regulation of Tau secretion. Thus, the authors proposed that Rab1 could be a therapeutic target to modulate Golgi dynamics and Tau secretion in AD [16]. In summary, GA fractioning is associated with Tau phosphorylation [71], pTau accumulation in NFT [72] and Tau secretion [16]. Hence, Rab1 GTPase regulation could modulate such neurodegenerative processes.

Regarding PD, dopaminergic neurons also display GA fragmentation. Specifically, dopaminergic neurons from the substantia nigra pars compacta that overexpress human α-syn exhibit GA fragmentation, which is reduced when overexpressing Rab1A [17]. Additionally, apart from rescuing GA fragmentation, Rab1A overexpression in dopaminergic neurons induced improvements in motor functions. Conversely, overexpression of the non-prenylable Rab1A (Rab1A-ΔCC) was not able to rescue GA from the fragmentation. This demonstrated the importance of Rab1A in the maintenance of the GA integrity, and consequently, in the control of motor functions [17]. These data suggest that Rab1A GTPase overexpression could be a therapeutic approach for this pathology.

A recent study has analyzed dopaminergic neurons from the substantia nigra of human patients with PD, and they have demonstrated that GA is fragmented and that the surviving neurons show a high overexpression of Rab1 GTPase [18]. The authors suggest that this overexpression of Rab1 could induce the GA fragmentation by two theoretical mechanisms proposed: (1) overexpressed Rab1 could alter ER–Golgi transport, therefore causing an imbalance in the GA; (2) Rab1 could be interacting with Golgin-84, which would be inducing the fragmentation [18]. Overall, there are discrepancies regarding the role of Rab1 in either inducing or preventing GA fragmentation in PD.

Apart from AD and PD, ALS is another neurodegenerative disease that presents GA fragmentation. The major cause for this seems to be the disturbances in the secretory pathway dependent on Rab1 [70]. Thus, Rab1 and its role in mantaining GA integrity is involved in different neurodegenerative diseases.

#### 2.1.3. Rab1 and the Control of the Autophagosome

Rab1 GTPase, along with other Rab GTPases such as Rab5, Rab7, Rab9A, Rab11, Rab23, Rab32, and Rab33B, participates in the formation of the autophagosome [73] at its beginning by recruiting the autophagy-related protein 9 (Atg9), a transmembrane protein responsible for transporting membranes to the phagophore, which is the structure preceding the formation of the autophagosome [74,75].

As previously mentioned, α-syn overexpression induces GA fragmentation. This leads to the dysregulation of autophagy in the SKNSH human neuroblastoma cell line, HeLa, HEK293 and M7-α-syn mice [20]. Winslow and colleagues described that α-syn alters the activity of the Rab1A/Atg9 axis. When silencing Rab1A and overexpressing α-syn, the Atg9 protein stopped localizing at a perinuclear position and passed to a diffuse distribution, resulting in a reduction in the autophagosome formation [20]. Thus, an increase in Rab1A activity could favor autophagy and therefore reduce the severity of the disease, as this mechanism could be used to recycle and eliminate protein aggregates.

### 2.2. Rab5

Rab5 plays an important role in endocytosis, being responsible for the fusion of endocytic vesicles coming from the plasma membrane to form early endosomes. By this mechanism, Rab5 regulates the internalization and the trafficking of membrane receptors [76].

The two GEFs described for Rab5 are Ras/Rab Interactor 3 (RIN3) and Rabex5. RIN3 is a member of the RIN family of GEFs, together with RIN1 and RIN2. All three have a Vps9 domain, which is the Rab5-specific GEF catalytic domain [77]. Regarding Rabex5, it is the best-understood member of the Vps9 domain-containing GEFs. Apart from its catalytic domain, Rabex5 contains a Rabaptin5 binding site, which is a Rab5 effector molecule. Thus, Rabex5 binds tightly to Rab5-regulated Rabaptin5, which in turn regulates Rabex5 GEF activity, forming a feedback loop [78].

Rab5 recruits Rabaptin5 in early endosomes, the latter being responsible for the docking and fusion of membranes [79]. Once activated, the Rabex5/Rab5/Rabaptin5 complex is localized in endocytic vesicles and early endosomes [79,80,81]. The three molecules work to stabilize active Rab5 once it reaches its targeted localization, forming a positive feedback loop that potentiates this pathway [38].

As the Rab5 signaling to Rabaptin5 [79] above described, Rab5 can signal through the PI3K hVPS34-p150 complex, which increases the levels of PI3P in early endosomes [25,82,83]. This PI3P permits the recruitment of the EEA1, another Rab5 effector molecule that regulates the docking of endocytic vesicles before their fusion with the early endosomes [84]. Moreover, hVPS34-p150 can activate a negative feedback loop by activating TBC1D2 GAPs, resulting in Rab5 GTPase inactivation [85]. The TBC domain-containing GAPs TBC1D3, RUTBC3, and USP6NL have been described as Rab5 GAPs [12,41].

The role of Rab5 in neurodegenerative diseases has been circumscribed to endosomal trafficking. In this regard, various studies have detected an increase in Rab5 activity in AD [12,22,86,87,88,89,90,91], as well as in murine models of PD [12,92,93].

In Huntington’s disease (HD), Rab5 also controls the motility of early endosomes. HD is caused by mutations in the huntingtin (Htt) protein, which is located on the GA and on vesicles. Htt forms a complex with Htt-associated protein 40 (HAP40) and serves as an effector molecule of Rab5 [94]. In HD, HAP40 is upregulated and Htt-HAP40 complex is disrupted. Consequently, the motility of the early endosomes is reduced [94]. Thus, Rab5 could be a therapeutical target to improve the endosomal motility in HD.

#### 2.2.1. Rab5 and APP Processing

The anomalies in endocytic trafficking are one of the main characteristics of AD, and according to Cataldo and collaborators, they precede the Aβ deposits [95]. A later study demonstrated that Rab5 overexpression can reproduce such endocytic anomalies by increasing the highly active processing of APP in endosomes [22]. The overexpression of Rab5 in murine cells induced endocytic changes related to AD, such as the presence of big endosomes similar to those observed in neurons from AD brains [22].

Furthermore, Rab5 overexpression increased by 2.5 times the levels of Aβ_1-40_ and Aβ_1-42_ secretion [22]. The authors also observed an increase in the βCTF levels. These βCTFs colocalize with early endosomes, suggesting a direct relationship between the endosomal pathway, βCTF generation, and Aβ production. Therefore, the endosomal anomalies observed in AD could be associated with the defects in APP proteolysis [22]. This suggests that Rab5 could be a therapeutic target due to its relevance in the control of APP processing and consequently, in Aβ_1-40_ and Aβ_1-42_ generation.

The role of βCTF in the recruitment of pleckstrin homology and phosphotyrosine binding domain- and leucine zipper motif-containing adaptor protein (APPL1) has also been described [91]. In endosomes, APPL1 stabilizes active Rab5-GTP, leading to a pathologic dysregulated endocytosis [91]. Taking into account the role of Rab5 in the endosomal pathway, Grbovic and collaborators defend that the dysregulations in the endosomes give rise to an increase in βCTF [22], and Kim and collaborators defend that βCTFs induce those endosomal dysregulations [91]. Additionally, shRNA silencing of BACE1 reverted the endocytic defects, suggesting that APP proteolysis could be the cause of the endocytic defects [96].

In conclusion, these studies point out a positive feedback loop in which the APP processing could lead to dysregulation of the endosomal pathway, and the defects in the endocytic pathway could in turn result in an increase in APP processing.

#### 2.2.2. Rab5 and Axonal Transport

In normal basal forebrain cholinergic neurons (BFCNs), the nerve growth factor (NGF) binds and activates the TrkA receptor at axonal ends. The NGF-TrkA complex is then internalized by endocytosis mediated by Rab5. The endosomes are transported in a retrograde direction through microtubules to the cell body, where the growth and differentiation signals are propagated to the nucleus [12].

In pathological conditions, there is an overactivation of Rab5 in BFCN neurons, which results in bigger early endosomes. These endosomes interfere in the retrograde axonal transport of NGF signals. Additionally, an increase in Rab5 activity can also affect motor proteins, altering axonal transport, and defects in the transport of trophic signals to the cell body lead to neuronal atrophy [12].

In this regard, the GEF RIN3 has been related to the overactivation of Rab5 in the transport of trophic signals [77,97]. Moreover, genome-wide association studies (GWAS) have linked RIN3 with the risk of developing AD [12,98,99,100]. However, it still needs to be clarified whether RIN3 function and expression are altered in AD and if other Rab5 GEFs underlie Rab5 over-activation in AD [12].

Nevertheless, there is another possible mechanism that could explain Rab5 overactivation. As previously mentioned, βCTF recruits APPL1 to the endosomes, which stabilizes Rab5-GTP. This complex leads to dysregulated endocytic pathways, as well as altered axonal transport [12,91].

Regarding PD, murine models constitutively expressing human α-syn have demonstrated the α-syn-dependent activation of Rab5 leading to dysregulation in Rab5 and dynein complex resulting in endosomal dysfunction. This could be the underlying mechanism that would explain the dysregulation in retrograde axonal transport and the consequent neuronal atrophy in PD [12,93].

### 2.3. Rab7

Rab7 GTPase regulates vesicular transport, specifically the late endocytic pathway [101]. It presents a fundamental role in the maturation of endosomes, in the transport of endosomes and lysosomes, in the fusion of late endosomes and lysosomes, and the lysosomal biogenesis [26,101,102]. Rab7 also participates in the traffic of autophagosomes [103]. Considering the importance of all these processes, Rab7 has been proposed as a therapeutic target for cancer [26] and neurodegeneration [104].

Rab7 activation is mediated by the GEF Mon1-Ccz1 [27,105,106]. The mechanism by which Mon1-Ccz1 mediates Rab7 activation consists its ability to be an effector molecule of Rab5 and interacting with PI3P in early endosomes [102,107]. This way, there is an exchange between Rab5 and Rab7 and the endosome passes from an early endosome to a late endosome [105,107]. On the other hand, the GAPs described for Rab7 are TBC1D2A, TBC1D5, TBC1D15, and EVI5-L [41].

Rab7-GTP in late endosomes and lysosomes can signal through its effector molecule the Rab-interacting lysosomal protein (RILP) [108]. RILP recruits dynein–dynactin motor complexes and consequently, the endosomes are transported towards the minus end of the microtubules [109]. The FYVE and coiled-coil domain-containing protein 1 (FYCO1) is another effector molecule of Rab7 that mediates the vesicular transport towards the plus end of microtubules [110]. Moreover, FYCO1 forms a complex with Rab7 and the LC3 protein, which is in charge of the maturation of the autophagosome [111]. Once this complex is formed, autophagic vesicles are transported towards the plus end of the microtubule [110].

Regarding the nervous system, both autophagy and the endolysosomal traffic governed by Rab7 have been associated with pathologies such as AD, PD, HD or Charcot–Marie–Tooth type 2B (CMT2B) [104,112]. Rab7 is involved in the traffic of toxic peptides such as Aβ vesicles [23] or Tau secretion in AD [29] and α-syn clearance in PD [30].

#### 2.3.1. Rab7 and Trafficking of Toxic Peptides

In AD, Aβ accumulation can be the consequence of a dysregulation in the APP processing, as well as a defect in the elimination of the toxic oligomers [113]. Therefore, the Rab5 and Rab7-controlled endolysosomal traffic are important for the clearance of toxic peptides such as Aβ. In this regard, studies in the N2a neuroblastoma mouse cell line, as well as in primary neuronal cultures from mice, have demonstrated that Aβ_1-42_ is internalized in Rab5-positive early endosomes at initial states and later, in Rab7-positive late endosomes [23]. These data suggest that the endocytic pathway is actively involved in the clearance and/or elimination of Aβ.

The overexpression of the dominant-negative forms of Rab5 and Rab7, unable to bind and transmit the signal through their effector molecules, inhibited the colocalization of these GTPases with Aβ_1-42_ monomers and oligomers in the endosomes [23]. This supports the involvement of these GTPases and endocytosis in Aβ clearance.

In fact, some studies suggest that the Rab5- and Rab7-mediated dysregulated endolysosomal pathway has toxic effects [24,87,88]. Post mortem AD brains have shown increased Rab5 and Rab7 protein levels [87,88]. Moreover, a study in primary neurons from the rat cortex has demonstrated that a Rab5- and Rab7-mediated active internalization of Aβ_1-42_ leads to neuronal death [24], and adding that the endocytosis general inhibitor phenyl arsine oxide (PAO) attenuated the toxicity. These results suggest that blocking Rab5- and Rab7-mediated endocytosis could be a therapeutic strategy to prevent neuronal death in AD [24].

As for Tau, the brains from patients with rapid progressive AD and 5XFAD mice brains exhibited increased Rab7A protein levels colocalized with pTau [28]. Moreover, Rab7A overexpression in primary cortical neurons and HeLa cells induced Tau secretion [29]. Conversely, Rab7A silencing, as well as the overexpression of its dominant-negative form, partially blocked Tau secretion [29]. All these data could mean that Rab7 dysregulation could contribute to Tau accumulation, as well as to the propagation of its toxic effects in AD [114].

#### 2.3.2. Rab7 and Endolysosomal Trafficking of Membrane Receptors

Endolysosomal pathway dysfunction has been related to PD, and genes that participate in this pathway have been related to this pathogenesis [115]. Lrrk, the homolog of the LRRK2 kinase in *D. melanogaster*, interacts with Rab7 in the membranes of late endosomes and lysosomes and has been shown to inhibit the Rab7-dependent perinuclear localization of lysosomes [116]. Conversely, the mutant form of Lrrk, the analog to the pathogenic LRRK2^G2019S^, promotes the perinuclear clustering of lysosomes. Thus, Rab7 and the LRRK2^G2019S^ could underlie the dysfunctional endolysosomal pathway in PD [116].

It has been described that LRRK2 regulates the Rab7-dependent endocytic traffic of the epidermal growth factor receptor (EGFR) [31]. The expression of the mutant LRRK2^G2019S^ caused a delay in early-to-late endosomal EGFR trafficking and a consequent delay in EGFR degradation. These defects were reverted by overexpressing the constitutively active form of Rab7 [31].

The ability of Rab7 to regulate the trafficking of receptors has already been used in therapeutic approaches for multiple sclerosis (MS) [33]. The overexpression of Rab7 can regulate the presence of Toll-like receptors (TLRs) and therefore control the inflammatory response [33]. However, Rab7 is not the only Rab GTPase that regulates the trafficking of receptors. Rab11, for instance, controls the TLR trafficking via the endosomes [117]. In this regard, the presence of specific single nucleotide polymorphisms (SNPs) in Evi5, a Rab11GAP, has been correlated to higher susceptibility for developing MS [118]. This suggests that Rab11 could be recycling TLR receptors, affecting innate immunity. More recently, Evi5 has been associated with MS [119] and it has been used as a marker for the disease [120]. These data invite one to explore Rab GTPases signaling regulation as an approach to promote the recycling of receptors in neurodegenerative diseases.

#### 2.3.3. Parkin/Rab7/RILP

Parkin is a ubiquitin E3 ligase associated with PD, as mutations in this enzyme are the second most common genetic risk factor for the development of the disease [121]. Rab7 K38 residue ubiquitination maintains Rab7 in an active form and consequently affects the endocytic traffic [32].

Experiments with primary fibroblast cultures from PD patients deficient of functional Parkin and in cells overexpressing the Rab7^K38R^ mutant that cannot be ubiquitinated demonstrated that in these situations, the Rab7 capacity of binding to its effector molecule Rab7-Interacting Lysosomal Protein (RILP) is diminished [32]. RILP is a Rab7 effector molecule involved in transducing the Parkin/Rab7 axis signaling. Specifically, RILP recruits dynein–dynactin motor complexes so that vesicles can be transported towards the minus end of the microtubules [108,109]. According to Song and collaborators, Rab7 dysregulation could be the main cause of endocytic alterations in Parkin^-/-^ cells. Moreover, these dysregulations of the Parkin/Rab7/endocytosis axis could contribute to the progression of the PD pathology [32].

#### 2.3.4. Rab7 and Autophagy

Rab7 in its active form can regulate the formation of the autophagosome, as well as its maturation and transport towards the microtubules [104]. The study of Rab7 and its role in autophagy could facilitate the development of strategies for the treatment of neurodegenerative diseases [104].

Rab7 is related to autophagy in CMT2B neurodegenerative disease. This pathology is caused by different missense mutations in Rab7 that lead to the reduced localization of Rab7 to autophagic compartments and decreased autophagy [8,34]. It is described that CMT2B is a direct consequence of Rab7 dysfunction, although it still needs to be clarified whether the pathology is a consequence of a reduction in the autophagic pathway due to Rab7 loss of function [8].

Regarding PD, studies with HEK293 and *D. melanogaster* α-syn^A53T^ demonstrated that Rab7 overexpression favors the clearance of α-syn aggregates [30]. Moreover, the authors identified that Rab7 localized in the neuromelanin granules in the human *substantia nigra* [30]. The Rab7/neuromelanin granules are autophagosome-like protective organelles. Rab7 participates in the biogenesis of these granules and the clearance of α-syn aggregates [30]. In addition, Rab7 overexpression in *D. melanogaster* rescued the phenotype and improved the locomotor deficits [30].

Nevertheless, Rab7 is not the only Rab GTPase described to control the α-syn clearance through autophagy. Recently, Rab27b has been shown to control the endolysosomal traffic and thereby the secretion and clearance of α-syn through autophagy [122]. Accordingly, the silencing of Rab27b by shRNA increased the intracellular levels of insoluble α-syn. Additionally, the post mortem brains of PD patients have shown increased protein levels of Rab27b [122].

Although they are not related to autophagic processes, other Rab GTPases also participate in the homeostasis of α-syn; whereas some of them favor the clearance of the aggregates, others favor their formation. For instance, Rab39B classically regulates the transport between the GA and the post-synaptic membrane. In PD, mutations in Rab39B have resulted in the loss of function of the GTPase and, consequently, in the dysregulation of α-syn homeostasis [123,124]. Conversely, PD patients have shown increased levels of Rab35, which promotes an augmented aggregation and secretion of α-syn^A53T^ [125]. Besides, primary cell cultures and in vivo experiments demonstrated that LRRK2-mediated Rab5 dysregulation induced severe neurotoxicity and the loss of dopaminergic neurons [57,58].

## 3. Arf GTPases in Neurodegeneration

Arf GTPases belong to a family of 29 members classified in different subfamilies: Arf1-6, Arf-like proteins (Arl), SARs, and Trim23 [9,126,127]. Arf GTPases are differentiated from Ras, Rho and Rab families as they possess an N-terminal extension of about 14 amino acids that can be covalently modified. In this regard, Arf GTPases can be N-myristoylated whereas Arl GTPases can be myristoylated, palmitoylated or acetylated [9].

Arf GTPases control cellular processes such as the bidirectional trafficking of membranes (secretion and endocytosis), metabolism of lipids, motility, division, apoptosis, and gene transcription [9,127]. However, their main role is the recruitment of coat proteins and complexes during vesicle formation in the membrane trafficking, particularly in the Golgi [9]. Thus, Arf GTPases, as well as their GEFs and GAPs, are localized in the plasma membrane, endosomes, lipid droplets, mitochondria, and lysosomes [9].

Like all GTPases of the Ras superfamily, the activity of Arf GTPases is regulated by GEFs, GAPs, and GDIs. In humans, 15 Arf GEFs have been described, and are classified in six families depending on their domains: GBF, BIGs, Cytohesins, EFA6/Psd, BRAG/IQSec and FBX [9]. All of them share in common the Sec7 catalytic domain [9,128,129]. Regarding the Arf GAPs, they are classified into 10 subtypes: ArfGAP1, ArfGAP2/3, ADAP1/2, SMAP1/2, AGFG1/2, GIT1/2, ASAP1-3, ACAP1-3, ARAP1-3 and AGAP1-11 [130,131,132]. They are characterized by their Arf GAP catalytic domain, although a family of proteins known as ELMOD have been demonstrated to possess GAP activity towards some Arf GTPases without having the Arf GAP domain [133,134,135]. Additionally, Arf GTPases can be regulated by post-translational modifications such as phosphorylation or ubiquitination [9].

Various Arf GEFs and GAPs have been described to play an important role in the nervous system. For instance, the Arf6 GAP, also known as ACAP3, has been shown to regulate neurite outgrowth in hippocampal neurons from mice [136]. Arf6 EFA6 GEF is involved in the arborization of dendrites and the formation of dendritic spines [137]. Moreover, mutations in the GEF BRAG1/IQSec2 have been associated with the nonsyndromic X-linked intellectual disability [138]. Another example is that mice with Schwann cell-specific GEF BIG1 knockout display reduced myelin thickness [139]. All of these studies demonstrate the fundamental importance of Arf GTPases, as well as their regulators in the nervous system.

With regard to Arf GTPases main effector molecules, they are components of vesicle coating, such as COP I, adaptor proteins (AP), GGA and MINT, which are the most studied [140]. COP I is a vesicle coating protein complex [141]. AP-1, AP-3, and AP-4 are clathrin adaptor proteins [9,140]. The GGAs participates in the TGN. Finally, MINTs interact with Munc18, a neuronal protein required for the exocytosis of synaptic vesicles [142].

**Figure 3:**
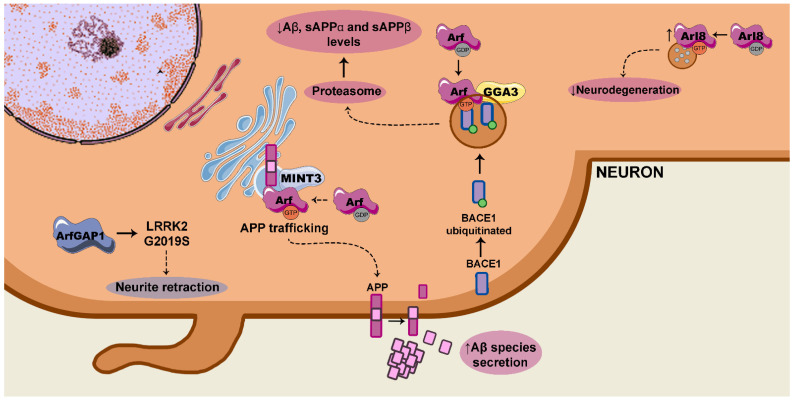
Scheme of the signalling pathways controlled by the small guanosine triphosphatases (GTPases) of the Arf family that are dysregulated in Alzheimer’s disease (AD) (purple) and Parkinson’s disease (PD) (blue). In AD, signalling pathways controlled by Arf are involved in amyloid precursor protein (APP) trafficking to plasma membrane and amyloid-β (Aβ) species secretion. Arf also controls Aβ species levels by favouring the proteasomal degradation of β-site APP cleaving enzyme 1 (BACE1). Arl8 blocks neurodegeneration mediated by Aβ. Regarding PD, ArfGAP1 silencing blocks the neurite retraction induced by LRRK2G2019S. sAPP: soluble APP; GGA3: Golgi-localized γ-ear containing Arf-binding protein 3.

Arf GTPases have been correlated to pathologies of the nervous system, such as ALS, retinal disease and Creutzfeldt–Jakob disease [143]. Moreover, Arf GTPases are associated with AD, as MINTs regulate APP trafficking [35] and the GGAs interact with BACE1 to control APP processing [144] (Figure 3).

### 3.1. Arf/MINT and APP Trafficking and Processing

MINTs are a family of three proteins that are specifically expressed in neuronal tissue. They are essential components for the fusion of neuronal synaptic vesicles [142,145].

MINT proteins directly bind to Arf-GTP and colocalize with APP in TGN regions [35]. MINT overexpression in HEK293 cells increased Arf GTPase-mediated APP protein levels [35]. Conversely, the siRNA-specific silencing of MINT3 in HeLa cells reduced APP protein levels. This demonstrated the Arf/MINT axis controls APP trafficking [35].

Another study demonstrated that MINT3 colocalized with APP in purified APP-containing vesicles in the SH-SY5Y neuroblastoma cell line [146]. The siRNA silencing of MINT3 impacted on APP trafficking, as well as its processing, inducing an increase in Aβ_1-40_ secretion [146]. Recently, a study in the N2a/APP695 mouse neuroblastoma cell line demonstrated that treatment with coconut oil reduced mRNA and protein levels of Arf1. Additionally, their results showed that Aβ_1-40_ y Aβ_1-42_ secretion levels were decreased [36]. All these studies suggest that Arf GTPase, along with its effector molecule MINT3, could be a therapeutic target to help regulate pathognomonic Aβ secretion in AD.

### 3.2. Arf/GGA/BACE1

The GGAs participate in the transport and sorting of proteins in the TGN [147]. One of the best studied functions of the GGAs is to direct the transport of ubiquitinated proteins to the endolysosomal pathway, as they possess ubiquitin-binding sites [148,149,150].

It has been described that GGA3 binds to the ubiquitinated BACE1 secretase to regulate its proteasomal degradation [151]. In this regard, the ectopic expression of GGA3 in GGA3-knock-out H4 neuroglioma cells blocked BACE1 accumulation, as well as Aβ_1-40_ secretion [151]. Therefore, the potentiation of the Arf/GGA3 axis activity could reduce BACE1 levels and consequently, Aβ secretion.

Nevertheless, a study demonstrated in HEK293 cells cotransfected with APP695 and BACE, that GGA1 sequesters APP together with BACE1 into the Golgi [152]. Consequently, BACE1 processes APP resulting in an increase in βCTF levels. Curiously, neither the intracellular levels nor the secretion of Aβ_1-40_ were increased. The authors conjectured that GGA1 sequesters the APP into the Golgi together with BACE1, where the β-cleavage takes place, and GGA1 also prevents these βCTF fragments from being transported and processed by the γ-secretase. In this way, although βCTF is increased, GGA1 would be negatively regulating its processing by the γ-secretase and, consequently, the generation of Aβ_1-40_ [152]. In the same line, another study in HEK293 and N2a cell lines showed that GGA overexpression resulted in a reduction in the secretion of the soluble APP alpha (sAPPα), sAPPβ, and Aβ. siRNA silencing of GGA reversed this effect [144].

### 3.3. Arl8 and Neuroprotection Against Aβ

Arl8 GTPase promotes pre-synaptic vesicular and endocytic macromolecules traffic towards the lysosomes [153,154]. Various effector molecules are recruited to lysosomal membranes by Arl8-GTP [155]. For instance, the HOPS complex is responsible for the fusion of compartments in the late endocytic pathway [153,156,157]. Another effector molecule described for Arl8 in mammals is the SKIP/PLEKHM2, which is a linker protein that recruits kinesin-1 to lysosomal membranes [155,158].

It has been described that silencing the expression of Arl8 in *C. elegans* neurons provokes the Aβ-mediated neurodegeneration [37]. On the contrary, the overexpression of Arl8 partially blocked this neurodegeneration. The authors demonstrated that the neuroprotective role of Arl8 depended on its state of activation. Constitutively active Arl^Q75L^ partially reduced the neurodegeneration, whereas the dominant-negative Arl^T34N^ did not have any protective effect [37]. The authors suggest that Arl8 could inhibit neurodegenerative processes through the activation of autophagy [37,159].

### 3.4. ArfGAP1/LRRK2

LRRK2 is a multidomain protein that presents kinase activity as well as GTPase activity [160], described to be controlled by ArfGAP1 in HEK293 and brain extracts from mice [161]. The ArfGAP1/LRRK2 regulation is reciprocal, as LRRK2 can phosphorylate ArfGAP1 and increase its activity. Moreover, ArfGAP1, apart from activating GTP hydrolysis, also increased LRRK2 kinase activity, suggesting that ArfGAP1 activity could be implicated in this kinase activation [161].

It is known that primary neurons from LRRK2^G2019S^ mice display neurite retraction. Stafa and collaborators rescued this phenotype by silencing ArfGAP1 [161]. Therefore, this GAP could be a possible therapeutic target for PD [161].

## 4. Future Perspectives

The purpose of the drugs used for the treatment of AD and PD is to enhance cognition and ameliorate the symptoms. Thus, the FDA-approved drugs for both diseases include cholinesterase inhibitors and N-Methyl D-Aspartate (NMDA) receptor antagonists [162]. However, the symptomatic treatments do not strike the origin and the progression of the disease. In this regard, some approaches are currently being studied to decrease Aβ production, reduce the aggregation or enhance its clearance on the one hand, and inhibit Tau phosphorylation on the other [162], and new dopaminergic drugs are currently being tested for PD [162].

Both diseases, AD and PD, share common features such as the generation and accumulation of toxic peptides. Nevertheless, a few studies are focusing on the role of membrane and vesicular trafficking in the generation, accumulation and clearance of those peptides. Specifically, the position of the Rab and Arf GTPases in these processes should be given more attention and targeting them could be a promising therapeutic approach.

One strategy could be modulating Rab and Arf GTPase activity depending on their state in the pathology. The expression of constitutively active or dominant-negative forms of these GTPases may be another alternative. For instance, constitutively active Arl^Q75L^ reduced Aβ-induced neurodegeneration in *C. elegans* [37]. Another example is the dominant-negative form of Rab7A, which partially blocked Tau secretion [29]. Moreover, targeting the expression by siRNA silencing techniques could serve as a therapeutic strategy. In fact, Rab7A silencing by siRNA has been proven to be effective in the reduction in Tau secretion too [29]. A recent study has hinted that modifying Arf GTPases could be a feasible approach. Coconut oil treatment in N2a/APP695 cells reduced Arf1 mRNA and protein levels, which resulted in a decrease in Aβ_1-40_ and Aβ_1-42_ secretion levels [36].

Additionally, targeting the PTMs that allow GTPases to be anchored to cellular membranes could be a promising approach in the case of Rab and Arf families, as they are key regulators of membrane and vesicle trafficking. This strategy has already been proven to be effective in other families of the Ras superfamily [5,163]. For instance, the inhibition of Rho family PTMs by lovastatin promotes myelin repair [163].

The regulation of GEFs, GAPs, and GDIs that control specific Rab and Arf GTPases could be an additional approach to treat neurodegenerative diseases. Lastly, peptides that interfere with protein–protein interactions between GTPases and their respective effector molecule is a promising alternative, as specific interactions can be inhibited without affecting the GTPase interaction with other effector molecules [5].

The problem lies in the fact that some Rab and Arf GTPases can sometimes have a neuroprotective role, whereas other times they can be neurotoxic. For instance, Rab5 overexpression has been shown to increase Aβ_1-40_ and Aβ_1-42_ secretion [22] and Rab7 seems to contribute to Tau secretion [29]. However, endolysosomal traffic controlled by Rab5 and Rab7 appears to favor the clearance of Aβ [23,24]. Another example is Rab1. Whereas Rab1 has been described to prevent GA fragmentation [16,17], it has recently been reported to possibly induce this fragmentation in neurons from human PD patients [18]. The presence of GTPases with opposite roles in neurodegenerative diseases further complicates the development of therapeutic approaches. The pathological state of activation in each specific case should be taken into consideration when focusing on small GTPases as therapeutical targets.

In addition, it is important to describe the whole signaling cascade controlled by each GTPase in each specific pathological condition before considering a GTPase as a therapeutic target. While many signaling cascades are described in neurodegeneration in the Ras and Rho families [5], pathways controlled by Rab and Arf families need deeper study. Thus, the description of the exact axis controlling each toxic response would help us to identify therapeutic objectives. Considering this, the field should advance in describing the precise signaling cascades controlled by Rab and Arf GTPases in neurodegeneration in order to detect potential targets. However, not only is it important to describe the signaling cascades, but identifying the location of the specific pool that is being activated in a cell is relevant too [5].

Furthermore, the implication of glial cells should not be disregarded. Most studies in Rab and Arf GTPases have been carried out in neuronal cells, ignoring the involvement of glial cells in the pathogenesis of neurodegenerative diseases. For instance, microglial cells present a high volume of membrane trafficking as they actively participate in the clearance of protein aggregates. Therefore, methods specifically targeting glial cells could be a promising therapeutic option.

Apart from emphasizing the search for therapies, studies should focus on the search for an early detection of neurodegenerative diseases. For instance, a liquid biopsy-based early diagnosis would improve the outcome of neurodegenerative diseases and researchers are currently trying to find biomarkers for the early detection [164].

However, if the biomarker needs to be present in blood, it has to be able to cross the blood–brain barrier [165]. Another problem is that the concentration of the biomarker in blood could be lower than in the cerebrospinal fluid; for instance, Aβ concentrations are 10-fold lower in plasma [165]. Thus, highly sensitive techniques would be required for the detection of biomarkers in blood. Despite these challenges, liquid biopsy-based diagnosis will soon be performed in the field of neurodegenerative diseases [164].

## 5. Conclusions

The Ras superfamily of GTPases has long been disregarded as potential players in neurodegenerative diseases. Recently, we reviewed the role of Ras and Rho families, as well as their regulatory and effector molecules, as potential participants in the pathogenesis of neurodegeneration [5]. When it comes to Rab and Arf GTPases, the field is less explored, and very few studies have associated these molecular switches with AD and PD. However, these studies are a clear indication that Rab and Arf families are involved in the pathogenesis of neurodegenerative diseases.

In a broad concept, Rab GTPases in physiological conditions are responsible for vesicular transport and membrane trafficking [38]. They control the integrity of the GA, the processing and trafficking of toxic peptides such as the APP, the axonal transport of proteins such as membrane receptors and autophagy. Regarding Arf GTPases, their main function is to control vesicle formation, although they are also regulators of the membranes bidirectional trafficking [9]. In this way, they manage the trafficking of proteins such as APP and BACE1.

We expect that future research will allow us to characterize the whole signaling cascades controlled by Rab and Arf GTPases in neurodegeneration, and this will hopefully facilitate the development of therapeutic strategies. However, it must be highlighted that most studies have been done in neuronal cells, ignoring the involvement of glial cells in the pathogenesis of neurodegeneration. Thus, the role of these GTPases in AD and PD should be studied not only in neurons but in the nervous system as a whole.

## Figures and Tables

**Table 1:** Role of Rab and Arf GTPases in neurodegeneration. Schematic table of Rab and Arf GTPases and their described role in neurodegenerative diseases in different models. Aβ: amyloid-β AD: Alzheimer’s disease; ALS: amyotrophic lateral sclerosis; APP: amyloid precursor protein; α-syn: α-synuclein; βCTF: β-cleavage C-terminal fragments; CMT2B: Charcot–Marie–Tooth type 2B; ER: endoplasmic reticulum; GA: Golgi apparatus; HD: Huntingon’s disease; MS: multiple sclerosis; PD: Parkinson’s disease; TLR: toll-like receptor.

GTPases	Pathology	Role	Model Used	References
*Rab1*	AD	Prevention of GA fragmentation Regulation of Tau secretion	Human Tau-expressing HeLa cells Primary cortical neurons from rat	[16]
	PD	Prevention of GA fragmentation Improvement of motor functions	Human α-syn-overexpressing dopaminergic neurons from the substantia nigra pars compacta	[17]
	PD	Possible induction of GA fragmentation	Dopaminergic neurons from substantia nigra of human PD patients	[18]
	PD	Rescue of α-syn-induced loss of dopaminergic neurons	*C. elegans* *D. melanogaster* Primary neurons from rat	[19]
	PD	Control of autophagy through Atg9	SKNSH HeLa HEK293 M7-α-syn mice	[20]
	ALS	Control of ER–GA transport Control of ER stress	N2a cells	[21]
*Rab5*	AD	Endocytic alterations Increase in βCTF and Aβ species secretion	Stably overexpressing human APP695 murine fibroblast-like L cells (L/APP)	[22]
	AD	Internalization of Aβ_1-42_	N2a cells Primary neurons from mice Primary cortical neurons from rat	[23,24]
	AD, PD	Alterations in the axonal transport of trophic signals and consequent neuronal atrophy	Basal forebrain cholinergic neurons Human α-syn expressing murine models	[12]
	HD	Control of motility of early endosomes	Primary human fibroblast cell lines Human postmortem HD brains	[25]
*Rab7*	AD	Internalization of Aβ_1-42_	N2a cells Primary neurons from mice Primary cortical neurons from rat	[26,27]
	AD	Colocalization with pTau	Rapid progressive AD human brains 5XFAD mice brains	[28]
	AD	Induction of Tau secretion	HeLa cells Primary cortical neurons from rat	[29]
	PD	α-syn clearance Improvement of locomotor deficits	HEK293 α-syn^A53T^ *D. melanogaster*	[30]
	PD	Reversion of defects in EGFR trafficking induced by mutant LRRK2	HEK239T cells HeLa cells Fibroblasts from LRRK2^G2019S^ PD patients	[31]
	PD	Dysregulation on vesicle transport in Parkin^-/-^ cells	Fibroblasts from Parkin^-/-^ PD patients	[32]
	MS	Regulation of TLR trafficking	Human dendritic cells	[33]
	CMT2B	Control of autophagy	HeLa cells Skin fibroblasts from CMT2B patients	[34]
*Arf*	AD	Control of APP trafficking through MINT	HEK293 cells HeLa cells	[35]
	AD	Aβ species secretion	N2a/APP695 cells	[36]
*Arl8*	AD	Blockage of Aβ-mediated neurodegeneration	*C. elegans*	[37]
